# Static balance and function in children with cerebral palsy submitted to neuromuscular block and neuromuscular electrical stimulation: Study protocol for prospective, randomized, controlled trial

**DOI:** 10.1186/1471-2431-12-53

**Published:** 2012-05-16

**Authors:** Soráia Kazon, Luanda A C Grecco, Hugo Pasini, João C F Corrêa, Thaluanna C L Christovão, Paulo de TarsoCamillo de Carvalho, Lilian Chrystiane Giannasi, Paulo R G Lucareli, Luis Vicente Franco de Oliveira, Afonso Shiguemi Inoue Salgado, Luciana M M Sampaio, Claudia S Oliveira

**Affiliations:** 1 Post Graduate Program in Reabilitation Sciences, Nove de Julho University, UNINOVE, Sao Paulo, Brazil; 2 Postdoctoral Fellowship of the Oral Biopathology Postgraduation Program- Unesp/Faculty of Dentistry, Sao Paulo, Brazil; 3 Physical Therapist, Student in Doctor’s Program in Biomedical Engeneering, Camilo Castelo Branco University, Sao Paulo, Brazil; 4 Post Graduate Program in Biophotonics Applied to Health Sciences, Nove de Julho University, UNINOVE, Sao Paulo, Brazil

## Abstract

**Background:**

The use of botulinum toxin A (BT-A) for the treatment of lower limb spasticity is common in children with cerebral palsy (CP). Following the administration of BT-A, physical therapy plays a fundamental role in potentiating the functionality of the child. The balance deficit found in children with CP is mainly caused by muscle imbalance (spastic agonist and weak antagonist). Neuromuscular electrical stimulation (NMES) is a promising therapeutic modality for muscle strengthening in this population. The aim of the present study is to describe a protocol for a study aimed at analyzing the effects of NMES on dorsiflexors combined with physical therapy on static and functional balance in children with CP submitted to BT- A.

**Methods/Design:**

Protocol for a prospective, randomized, controlled trial with a blinded evaluator. Eligible participants will be children with cerebral palsy (Levels I, II and III of the Gross Motor Function Classification System) between five and 12 years of age, with independent gait with or without a gait-assistance device. All participants will receive BT-A in the lower limbs (triceps surae). The children will then be randomly allocated for either treatment with motor physical therapy combined with NMES on the tibialis anterior or motor physical therapy alone. The participants will be evaluated on three occasions: 1) one week prior to the administration of BT-A; 2) one week after the administration of BT-A; and 3) four months after the administration of BT-A (end of intervention). Spasticity will be assessed by the Modified Ashworth Scale and Modified Tardieu Scale. Static balance will be assessed using the Medicapteurs Fusyo pressure platform and functional balance will be assessed using the Berg Balance Scale.

**Discussion:**

The aim of this protocol study is to describe the methodology of a randomized, controlled, clinical trial comparing the effect of motor physical therapy combined with NMES on the tibialis anterior muscle or motor physical therapy alone on static and functional balance in children with CP submitted to BT-A in the lower limbs. This study describes the background, hypotheses, methodology of the procedures and measurement of the results.

**Trial registration:**

RBR5qzs8h

## Background

Posture control is fundamental to the efficient performance of all activities of daily living and is a complex process that depends on the integration of vision, vestibular and peripheral sensations, commands of the central nervous system and neuromuscular responses, particularly muscle strength and reaction time [1-3]. The control of an erect posture requires the adaptation capacity of motor responses to the mutable demands of the task at hand as well as the environment and the body itself [4].

Postural stability is defined as the ability to maintain and control the center of mass of the body within the support base in order to prevent falls and control desired movements [5]. Oscillations occur due to the difficulty in maintaining the segments of the body aligned on a small base (the feet) [6]. Currently, there are a number of tests for measuring balance to obtain further information on posture deficits in a static position [1,5]. While there are easy-to-use functional scales for assessing posture control, advanced laboratory systems provide more detailed information on both static and dynamic balance [7]. The use of a balance platform is an easy, effective method commonly employed in balance analysis laboratories for assessing postural balance with regard to oscillations from the center of pressure in the anterior-posterior and medial-lateral directions [8].

Deficits in posture control have been identified as the greatest limitation to the motor development of children with CP [9,10]. CP refers to permanent, but mutable motor development disorders stemming from a primary brain lesion, leading to secondary musculoskeletal alterations and limitations in activities [11]. Motor impairment is the main alteration in children with CP, with consequent alterations in body biomechanics [12-14].

Children with CP are currently classified based on their degree of functional independence with regard to gross motor functions. The Gross Motor Function Classification System (GMCFS) for Cerebral Palsy [15] classifies children on five functional levels according to age. Children with motor problems classified on Level I can generally walk without restrictions, but tend to be limited with regard to more advanced motor skills. Those classified on Level II exhibit gait limitation in the outdoor environment. Those classified on Level III need assistance for locomotion. Those classified on Level IV execute manual activities with limited success and require continual supervision. Children classified on Level V generally have a very limited capacity to move, even with the use of assistive technology [15,16].

Spasticity is the most common aspect of CP, affecting approximately 80% of cases [17]. Spasticity is considered one of the main causes of muscle impairment as well as impaired motor function, gait and posture control [18]. Spastic diplegia is a very common form of CP, with a wide range of ambulatory outcomes, and is most frequently accompanied by ankle spasticity [19]. Abnormalities, such as excessive plantar flexion of the ankle, excessive flexion of the knees associated with valgus and an increase in the adduction and internal rotation of the hips may explain the difficulties individuals with CP have in maintaining both static and dynamic balance [20,21].

The use of botulinum toxin A (BT-A) is recommended for the treatment of spasticity in ambulatory children with CP to improve motor function and slow down the development of fixed deformity with the need for surgical intervention [22]. BT-A is currently widely used for a localized reduction in muscle tonus with minimal side effects [23]. BT-A causes chemodenervation and muscle relaxation by disrupting the release of acetylcholine into the presynaptic cleft of the neuromuscular junction [24]. The effect of BT-A begins within two to three days after infection, reaching a maximal effect at two weeks. The reduction in muscle tone lasts an average of three months, with a gradual increase in tone and gain in muscle strength occurring after this time [25,26].

The selective reduction in muscle tone allows an improvement in muscle control and balance, resulting in a greater range of motion in the joints and the potential for strengthening the antagonist muscle [27]. Muscle strengthening in children with CP poses a challenge to physical therapists due to the lack of muscle selectivity necessary for a specific strengthening program. Electrical stimulation has been proposed as a potentially useful modality for muscle strengthening in this population. However, the clinical use of this modality remains the subject of debate [28]. Recent experimental studies have found that the use of neuromuscular electrical stimulation (NMES) following the administration of BT-A was beneficial in the treatment of spasticity, improving muscle function and gait parameters [1,29] in patients with CP. NMES is a safe application of electrical current that stimulates the injured neuromuscular system in an attempt to reacquire or improve function [30].

In recent years, a large number of studies involving the treatment of spasticity and the analysis of static balance have been published, with special attention given to motor impairment in patients with CP in the quest for an approach to functional rehabilitation in this population [31]. However, the combination of BT-A, physical therapy and NMES has not been analyzed with regard to static and functional balance in children with CP.

## Methods/Design

### Primary objective

The primary aim of the proposed study will be to investigate the effect of NMES on dorsiflexor muscles in combination with motor physical therapy on static balance (oscillations from the center of pressure in the anteroposterior and mediolateral directions) in children with CP submitted to BT-A in the lower limbs in order to diminish spasticity.

### Hypothesis 1

NMES in the ankle dorsiflexor muscles in combination with motor physical therapy is more effective than physical therapy alone in improving static balance in children with CP submitted to BT-A in the lower limbs.

### Secondary objectives

The secondary objective will be to investigate the effect of NMES in the ankle dorsiflexor muscles in combination with motor physical therapy on functional balance, as assessed by the Berg Balance Scale, in children with CP submitted to BT-A in the lower limbs.

### Hypothesis 2

NMES in the ankle dorsiflexor muscles in combination with motor physical therapy is more effective than physical therapy alone in improving functional balance in children with CP submitted to BT-A in the lower limbs.

### Study design

**Figure 1:**
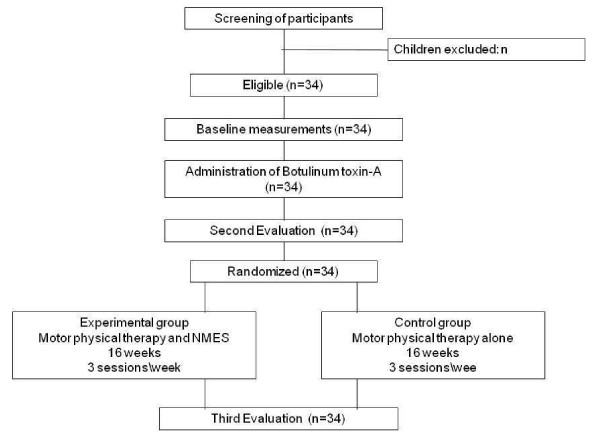
Flowchart representing the study design.

A prospective, analytical, controlled, randomized, two arms, blinded study will be carried out (Figure 1). The protocol for this study is registered with the Brazilian Registry of Clinical Trials - ReBEC RBR-994XFS.

### Ethical considerations

The present study complies with the principles of the Declaration of Helsinki and the Regulating Norms and Directives for Research Involving Human Subjects formulated by the Brazilian National Health Council, Ministry of Health, established in October 1996. The study received approval from the ethics committee of the Universidade Nove de Julho (Sao Paulo, Brazil) under protocol number 200903/2008. The participating institutions have provided a declaration of participation. All guardians agreeing to the participation of their child will do so by signing an informed consent form. The participants will be allowed to abandon the study at any time with no negative repercussions.

### Study sample and recruitment

Individuals with CP will be recruited from the physical therapy clinics of the Universidade Nove de Julho, Fisiovale and Centro de Neurocirurgia Pediátrica, Sao Paulo, Brazil. The participants will be recruited and selected based on the following eligibility criteria:

Inclusion criteria

Age between five and 12 years

Spastic, diplegic cerebral palsy

Motor function classified as Level I, II or III by the GMCFS

Independent ambulation with or without the need for a gait-assistance device (walker or crutches)

Dynamic equinus with indication for neuromuscular block with BT-A in the triceps surae

Availability for physical therapy twice a week for four months

The following inclusion criteria will also apply to one group of children: 1) degree of cooperation compatible with the administration of NMES and 2) tolerance to NMES at a motor threshold level (visible muscle contraction).

Exclusion criteria

Neurological or orthopedic conditions unrelated to cerebral palsy

Orthopedic surgery on the lower limbs in the 12 months prior to selection

Surgery scheduled during the period of the study

Equinus not reducible to neutral (ankle at 0°) or incompatible with the use of orthoses following the application of BT-A in the triceps surae

### Sample size

The calculation of the sample size was performed in a pilot study involving 12 children, in which there was an 11.9 reduction in oscillation from the center of pressure in the anteroposterior direction. Using a standard deviation of 10.6, α = 0.05 and 90% power, the sample is estimated as 17 children.

### Randomization

After fulfilling the eligibility criteria, undergoing the initial evaluation and receiving the BT-A, the participants will be randomly distributed into a experimental group (motor physical therapy and NMES) and a control group (motor physical therapy alone). The randomization numbers will be generated using a randomization table at a central office.

### Allocation concealment

A series of numbered, sealed, opaque envelopes will be used to ensure confidentiality. Each envelope will contain a card stipulating to which group the child will be allocated.

### Equipment

A duly calibrated mechanical scale (Welmy brand) with a precision of 0.1 kg and 0.1 cm will be used to determine body mass and height. The individuals will remain barefoot in a standing position on the center of the scale platform. A pressure plate (Fusyo model, Medicapteurs brand) with 2300 resistive sensors will be used for the assessment of static balance. This device measures oscillations in the center of pressure (COP) and contact time between the feet and the surface of the platform. The acquisition frequency will be 40 Hz. The data will be recorded and interpreted using the Fusyo Analysis software program. The Endophasys electrical stimulator (KLD Biosistemas) will be used for the electrical stimulation, which is made up of four 80 ma output channels, with a frequency of 0 to 200 HZ and pulse duration of 0 to 400 μsec.

### Intervention

#### Botulinum toxin-A

All participants will be submitted to the administration of BT-A in the lower limbs, performed by a single specialist in physical rehabilitation. The injected muscles will be determined based on the assessment of the specialist during the screening of the participants. The muscles to be injected are the gastrocnemius (lateral and medial), hip adductors and/or hamstrings, unilaterally for hemiplegic children and bilaterally for diplegic children. Body mass and application site will be considered for the calculation of the maximal dosage per patient. The maximal dose of BT-A (BOTOX, Allergan, Brazil) will be between 6 and 12 U/Kg of body mass, with a maximal possible dose of 200 U in compliance with the Brazilian Ministry of Health. Each vial of BT-A will be reconstituted with 3 to 5 mL of saline solution (NaCl 0.9%). The localization of the belly of the muscle will be determined with the children in ventral decubitus. Asepsis of the skin will be performed with 10% alcohol and none of the participants will receive sedation.

### Evaluation and follow-up

The children in both groups will be evaluated by two physical therapists experienced in the evaluation procedures and blinded to which group each child belongs. Three evaluations will be carried out:

Evaluation 1 One week prior to the administration of BT-A

Evaluation 2 One week following the administration of BT-A

Evaluation 3 Four months following the administration of BT-A (end of intervention protocol)

### Spasticity evaluation

Spasticity will be assessed using the Modified Ashworth Scale and Modified Tardieu Scale. The Modified Ashworth Scale measures the intensity of hypertonia, quantifying it as 0 (normal tonus) to 4 (rigidity) based on the degree of resistance the tested muscle offers to passive movement performed by the examiner [32,33]. The Modified Tardieu Scale is considered a clinically valid measure for the assessment of spasticity. It is an ordinal scale in degrees that measures the range of abrupt movement, which elicits hypertonia, and slow movement, which inhibits it. The measure of spasticity is obtained when a joint is moved as fast as possible through its range of movement (V3 velocity) and the angle of "catch" elicited is measured using a goniometer, which is called R1. The difference between the angle of "catch" (R1) and the full passive range of motion (R2) reflects the potential range available in the joint if spasticity is eliminated examiner [34-36]. The muscles to be evaluated are the hip adductors, hamstrings and triceps surae, bilaterally.

### Static balance

Static balance will be evaluated using a pressure platform (Medicapteurs Fusyo) This device measures oscillations in the center of pressure (COP) and contact time between the feet and the surface of the platform. The acquisition frequency will be 40 Hz. The children will be placed in an orthostatic position, barefoot, with arms alongside the body and eyes focused on a point marked at a distance of one meter positioned at the height of the glabella of each child. The evaluation will be carried out under two conditions for 30 seconds each: eyes open and eyes closed. The data will be recorded and interpreted using the Fusyo Analysis software program.

### Functional balance

The Berg Balance Scale will be used for the assessment of functional balance. This is a simple 14-item measure that addresses the performance of functional balance common to daily living. Each item has a five-option ordinal scale ranging from 0 to 4 points, with a maximal overall score of 56. The points are based on the time in which a position is maintained, the distance an upper limb is able to reach in front of the body and the time needed to complete the task. Execution time is approximately 30 minutes. The children will perform these tasks dressed, but barefoot [37,38].

### Standardized physical therapy program

The standardized program proposed by Ibrahin et al. (2007) will be used for the treatment of all children in both groups. One-hour sessions will be held three days a week on non-consecutive days. The protocol will last four months beginning with the administration of BT-A and will not undergo any alterations in this period. The program will consist of the following:

Muscle stretching for all muscles that can be stretched, especially those submitted to BT-A.

Use of a ankle-foot positioning orthosis for the correction of the deformity in plantar flexion of the ankle and to maintain the length and elasticity of the ankle muscles.

Gait training exercises, stressing the action of the ankle dorsiflexors, with different obstacles placed in the walking path; training in going up and down stairs.

### Neuromuscular electrical stimulation

The tibialis anterior muscle will be selected (bilaterally), with surface electrodes (5 cm X 5 cm) positioned on the motor point of the muscle. The following are the NMES parameters: frequency of 30 Hz, pulse from 300 μs [39], five seconds of ramp up, five seconds of maintenance, five seconds of ramp down and 10 seconds of rest, with the total application time of 10 minutes. The intensity will be set as high as the participant can tolerate [40]. A visible contraction will be produced in the tibialis anterior muscle, with the child seated with knees flexed, feet supported and ankles at 0 degrees. During the intervention protocol, the intensity of the NMES will be increased to maintain sufficient contraction in order to generated ankle dorsiflexion. The NMES sessions will be held after the physical therapy sessions (three days a week on non-consecutive days).

### Statistical analysis

The results will be expressed as mean values and 95% confidence intervals. The Shapiro-Wilk test will be used to analyze the homogeneity of the variances. If homogeneity is confirmed, one-way ANOVA will be used in the inter-group analyses and repeated-measure ANOVA will be used in the intra-group analyses. If homogeneity is not confirmed, the Kruskal-Wallis test will be used in the inter-group analyses and Friedman’s test will be used in the intra-group analyses.

## Discussion

This paper offers a detailed description of a randomized, controlled, blinded, clinical trial aimed at demonstrating the effect of NMES on the dorsiflexors of the ankle following the administration of BT-A in the triceps surae in ambulatory children with diplegic spastic cerebral palsy. The results will be published and contribute evidence regarding the use of NMES on this population.

## Abbreviations

BT-A: botulinum toxin type A; CP: cerebral palsy; NMES: neuromuscular electrical stimulation.

## Competing interests

The authors declare that they have no competing interests.

## Authors’ contributions

All authors contributed to the conception and design of the study. CSO and SK provided the idea for the study, established the hypothesis and wrote the original proposal. SK and CSO significantly contributed to drafting of this paper, while LACG, HP, JCFC, TCLC, PT, LCGM, PRGL, LVFO, ASIS and LMMS were involved in critically revising the manuscript. This protocol paper was written by SK, LACG and CSO with input from all co-authors. All authors read and approved the final manuscript.

## Pre-publication history

The pre-publication history for this paper can be accessed here:

http://www.biomedcentral.com/1471-2431/12/53/prepub
